# Improve teaching with modalities and collaborative groups in an LMS: an analysis of monitoring using visualisation techniques

**DOI:** 10.1007/s12528-021-09289-9

**Published:** 2021-07-13

**Authors:** María Consuelo Sáiz-Manzanares, Raúl Marticorena-Sánchez, Juan José Rodríguez-Díez, Sandra Rodríguez-Arribas, José Francisco Díez-Pastor, Yi Peng Ji

**Affiliations:** 1 Departamento de Ciencias de La Salud, Facultad de Ciencias de la Salud, Universidad de Burgos, Paseo de Comendadores s/n, 09001 Burgos, Spain; 2 Departamento de Ingeniería Informática, Escuela Politécnica Superior, Avda. Cantabria s/n, 09006 Burgos, Spain

**Keywords:** Online project-based learning, Visualisation techniques, Machine learning techniques, Monitoring students, Self-regulated learning, Heat map

## Abstract

Monitoring students in Learning Management Systems (LMS) throughout the teaching–learning process has been shown to be a very effective technique for detecting students at risk. Likewise, the teaching style in the LMS conditions, the type of student behaviours on the platform and the learning outcomes. The main objective of this study was to test the effectiveness of three teaching modalities (all using Online Project-based Learning -OPBL- and Flipped Classroom experiences and differing in the use of virtual laboratories and Intelligent Personal Assistant -IPA-) on Moodle behaviour and student performance taking into account the covariate "collaborative group". Both quantitative and qualitative research methods were used. With regard to the quantitative analysis, differences were found in student behaviour in Moodle and in learning outcomes, with respect to teaching modalities that included virtual laboratories. Similarly, the qualitative study also analysed the behaviour patterns found in each collaborative group in the three teaching modalities studied. The results indicate that the collaborative group homogenises the learning outcomes, but not the behaviour pattern of each member. Future research will address the analysis of collaborative behaviour in LMSs according to different variables (motivation and metacognitive strategies in students, number of members, interactions between students and teacher in the LMS, etc.).

## Introduction

Nowadays, the teaching–learning process is increasingly carried out in online or blended learning environments, reducing the use of the purely face-to-face (F2F) modality. This situation has increased with the crisis due to COVID-19 (Sáiz-Manzanares et al., 2020b). Therefore, the way of learning and teaching is changing, since a high percentage of teaching is done through Learning Management Systems (LMS) (Sáiz-Manzanares et al., 2020b). One of the challenges in these environments is to analyse the development of cooperative learning among groups of students. This challenge is very important in teaching designed from a constructivist methodology such as Project-based learning (PBL). The PBL methodology focuses on the development of critical thinking, encourages creativity and the resolution of tasks specific to the degree in the future graduate. Moreover, collaborative work is one of the skills required by today's society and is recognised by organisations such as the Organisation for Economic Cooperation and Development (OECD) (OECD, 2019) and by the European Commission in the 2030 Agenda for education. The next section will deal with aspects related to active teaching methodology such as PBL applied in LMS environments in collaborative groups and the analysis of logs through Machine Learning and visualisation techniques during the monitoring of students throughout the teaching–learning process. The digital transformation involved in tackling this methodological change in teaching requires the acquisition and use of digital skills by the agents involved (teaching staff and students). This transformation was planned at the beginning of 2020 in a progressive development. However, the SAR-CoV-2 pandemic accelerated the acquisition of these strategies and their implementation in different professional, educational and social contexts (García-Peñalvo, 2021). Nevertheless, this supervening need does not imply that institutions and citizens were prepared to face the challenge. Although a huge effort has been made, especially in higher education institutions, it has become clear that there are gaps in digital strategies among both teaching staff and students. This fact points to the need to address the challenge of digital transformation with concrete training proposals. These include changes in teaching instruction (teaching style) and in the way students learn (learning style) (Cabero-Almenara and Llorente-Cejudo, 2020). Similarly, the feedback that the virtual learning environment must offer to the teacher and the student involves increasing the use of visualisation tools for the teaching–learning processes in the LMS (Álvarez-Arana et al., 2020). In recent years, the use of these Learning Analytics tools has been boosted in order to be able to easily analyse the large volume of data recorded in LMSs (Duin & Tham, 2020). These resources will make it possible to detect the learning patterns of each user and, depending on the learning results, propose the most appropriate curricular aids for the student to achieve the deepest and most effective learning possible (García-Peñalvo, 2020a). Therefore, it is important to include dashboards in virtual platforms that provide teachers with information to monitor their students' learning patterns in real time (Verbert et al., 2013). All of this opens up a new challenge, which is the integration of data in educational contexts in higher education and its processing through ecosystems or holistic environments for interpretation (Vázquez-Ingelmo et al., 2021). Such ecosystems entail institutional decision-making aimed at a necessary change already initiated by the health crisis situation (García-Peñalvo, 2020b; García-Peñalvo and Corell, 2020). All these aspects will be addressed in more depth below.

## Background

### Project-based learning, flipped classroom, and self-regulated learning in virtual environments

PBL is a teaching–learning method that is based on constructivism (Hmelo-Silver, 2004). It can be defined as student-centred instruction that occurs over an extended period of time during which students select, plan, research and create a product, a presentation or a development that answers a research question or problem (Holm, 2011). PBL focuses on the teaching–learning process, on the interaction of teacher and students, on the construction of deep learning from the design of tasks based on practical research and directed through reflection questions. All of this is based on continuous formative feedback in a collaborative learning framework (Doise et al., 1975). Current studies (Lajoie et al., 2015) indicate that an important factor for the successful development of PBLs is the use of interactive platforms such as LMSs, in this case the PBL is called Online Project-Based Learning (OPBL) (Yilmaz et al., 2020). Such environments are tools that facilitate co-regulation and the development of metacognitive strategies during task solving (Bannert et al., 2015). Therefore, LMSs enable the design and implementation of structured programmes for the development and activation of metacognitive strategies during learning. This fact facilitates the development of Self-regulated learning (SRL) (Bannert, et al., 2014), as they structure the necessary sub-goals in a stepwise manner, in order to achieve deep and successful learning from consecutive approaches to the goal. The reason is that the use of self-planning (Sáiz-Manzanares & Montero-García, 2015), monitoring and self-assessment strategies will increase self-awareness (Cloude et al, 2019). This is because the teaching–learning structure developed in LMSs based on OPBLs can activate students' prior knowledge through enquiry questions implicit in the development of OPBLs (Brand et al., 2019). Furthermore, LMSs allow for continuous monitoring and adaptation to the student's learning pace, which increases the use of metacognitive and motivational strategies during the learning process (Cloude et al., 2019). All of which will facilitate the generalisation of metacognitive skills to achieve learning objectives (Wiedbusch et al., 2021). Therefore, in this environment, collaborative student work in small groups can be implemented, which will facilitate the work dynamics within the PBL methodology (Shanmuganeethi et al., 2020). All this will make sustainable education possible by making profitable use of the resources that the teacher implements on the platform, as these resources will be used according to the students' learning styles (Sáiz-Manzanares et al., 2021a). In short, higher levels of performance, coupled with higher quality of learning by students, are achieved when students mobilize metacognitive skills and deep approaches in their learning process (Bártolo-Ribeiro et al., 2020). Especially in higher education, when it requires greater autonomy of students, the capacities for regulating cognition and learning are fundamental to assure academic achievement (Valadas et al., 2017). Recent research also shows that the use of hypermedia resources facilitates the development of deeper and higher quality learning (De Kock, 2016), as systematic planning through interactive platforms, where both audio and video elements are additionally combined, facilitates the development of metacognitive strategies during task solving. These platforms set the objective, the planning in the development of the resolution, the evaluation of the resolution steps and the evaluation of the final result from a respect for the student's learning pace (Sáiz-Manzanares et al., 2019a). Working from the OPBL methodology combined with the feedback resources that LMSs include further increases motivation and autonomy in student learning (Chen et al., 2020). However, this is a complex and difficult process to measure (Zhang et al., 2019). Recent research (Aikina & Bolsunovskaya, 2020) has found that the most important factors for increasing motivation are: automatic checking of exams, the possibility to publish news and additional learning material, setting individual assignments, organising collaborative learning online and having analytics to track student behaviour in LMSs similar to Moodle (Modular Object-Oriented Dynamic Learning Environment). Current research in this field is directed towards testing the type of relationships that are established within collaborative groups during the resolution of OPBLs. It has been found that cooperation based on a vertical structure in which one of the members of the collaborative group guides the work process is the most effective (Yilmaz et al., 2020). In this framework, the use of Flipped Classroom experiences has also been shown to be a very effective resource for enhancing SRL and increasing motivation (Noroozi et al., 2019) and the effective development of OPBL, as it improves the use of metacognitive strategies of self-planning and self-assessment (Yoon et al., 2021). On the other hand, the teacher's interpretation of the results of the learner interaction in the LMS requires the use of log analysis and interpretation systems. Possible resources include the use of visualisation and data mining techniques throughout the learning process. These tools will facilitate the detection of the learner at risk in each of the collaborative groups throughout the learning process, therefore these aspects will be discussed below. Likewise, recent study shows that the use of hypermedia resources facilitates the development of deeper and higher quality learning (De Kock, 2016), since systematic planning through interactive platforms, in which both audio and video elements are additionally combined, facilitates the development of metacognitive strategies during the resolution of tasks.

### Learning management system relationship with self-regulated learning and metacognition: analysis using educational data mining techniques

Teaching work in LMSs within eLearning or Blended Learning environments is a practice that has been developing more and more frequently over the last decade. This has increased over the last year due to the COVID-19 pandemic. This practice refers to both formal and non-formal concepts of teaching and focuses on the importance of the human factor and interaction as an essential element in the learning process (García-Peñalvo and Seoane-Pardo, 2015). Nevertheless, the only use of LMS does not guarantee better learning outcomes (Agredo-Delgado et al., 2020). Such use is conditioned on the one hand by the teaching design and on the other hand by the type of feedback that the design includes on the evidence of learning. Recent studies (Park & Jo, 2017) have found significant differences in learning outcomes according to the teachers’ teaching style and the learning style of students (Sáiz-Manzanares et al., 2021a). Another relevant aspect facilitated by LMSs is the early detection of at-risk students, Strang (2016) analyses the relationship between the use of LMSs and students' learning patterns on the platform (Sáiz-Manzanares et al., 2021b). In this line, regression analysis techniques, among others, make it possible to detect successful and risky behavioural patterns. These patterns explain up to 52% of the variance in learning outcomes. These studies are supported by the use of Data Mining (DM) techniques. The learning behaviours that have been considered referential for successful learning, among others, are (Cerezo et al., 2016):

- The time students spend on tasks.
- The time spent working on theoretical content.
- Results in self-assessment tests (quiz efforts).
- Time spent in forum discussions.
- The quality of the discussions in the forums (type of message and length of the message).
- Time spent analysing the feedback given by the teacher.
- The frequency of use of the LMS.
- Contribution to content creation.
- The files viewed.
- The delivery time of the activities.

### Logs, learning analytics (LA) and educational data mining (EMD)

When the various participants in the learning process interact through an LMS, a series of logs or log files are generated that capture each of the interactions. These logs can be analysed. The use of DM will allow patterns to be isolated or new information to be extracted from the analysis of large data sets. When these techniques are used with data related to learning, we talk about Learning Analytics (LA) or Educational Data Mining (EDM) techniques. These concepts are closely related although they are not the same. LA focuses more on understanding the learning process (Agudo-Peregrina et al., 2014), specifically, these techniques investigate, among others, the answers to the following questions:

(1) Which data to analyse (what). The information recorded in the LMS is overwhelming, that is why it is necessary to detect data analysis patterns. (2) For whom this information is provided (who). It is important to discriminate the target group of the analysis (students, teachers, tutors/mentors, educational administrators, etc.). Students will be interested in more effective learning spaces. Teachers will be interested in how to make their teaching practices more effective and to offer the support their students need, and institutions will be interested in detecting students at risk in order to increase success rates and avoid drop-out. For all these reasons, it is increasingly important to implement tools within LMSs that offer data analysis so that teachers, who are not experts in the application of EDM techniques, can understand and clarify the different situations that occur during the learning process. These techniques provide aim-oriented feedback that allows the user of the platform to reflect on their actions and guide them in their decision making. (3) Why information is provided. There are different objectives depending on the user role. LAs include monitoring analysis, i.e. tracking students in order to generate reports for the teacher and/or the institution. This information will help the teacher to evaluate the learning process in order to improve the learning environment and offer help to the student in order to increase the results. As well as the prediction of the student's knowledge and learning results in order to detect the student at risk and provide him/her, if needed, with the necessary help to achieve effective learning. In addition to implementing tutoring (learning guidance process) and mentoring (concrete plan of personalised help in planning and supervision issues and preparation of new challenges specifically for each student according to their needs), assessment and feedback (facilitates self-assessment processes that allow the student to succeed in learning). Intelligent feedback reports are produced and provided to both the teacher and the learner. 4) How information is provided. Methods for detecting hidden patterns of learning in LMSs can be done through statistical methods, data visualisation methods and DM techniques (Einhardt et al., 2016). The former in LMSs allow the extraction of reports based on teacher and learner interaction on the platform (online time, total number of visits, number of visits per page, distribution of the visits over time, frequency of replications, etc.). The most commonly used statistics are means (M) and standard deviations (SD). The second ones offer user-friendly reports on data distribution (e.g. heat maps, bar area charts, histograms, scatter plots, etc.). And the third ones can be supervised (classification and regression) and unsupervised (clustering) learning DMs and data association rules. All of them can provide information about models (Slater et al., 2017). In short, the EDM technique is multidisciplinary, converging techniques of algorithm construction, Artificial Neural NetWorks, instance-based learning, Bayesian learning, etc. These techniques can use different analysis procedures (Arnaiz-González et al., 2016) which can be grouped into clustering techniques, outlier detection techniques, association rule mining and sequential pattern mining and text mining (Romero and Ventura, 2007). Therefore, the use of one or the other will depend on the objectives of the task analysis. However, researchers in this field seek to predict the results in order to provide particular recommendations in each case. Regarding LMSs, one of the most widely used is Moodle, which allows the use of different resources for different learner profiles (individual and/or group) and teacher profiles. Moodle also makes it possible to carry out different learning activities and actions (discussion forums, questionnaires, workshops, wikis, access to repositories, etc.) and to use innovative teaching methodologies such as OPBL. The interactive behaviours that can be analysed on this type of LMS (Yücel & Usluel, 2016): student–student interaction, student–teacher interaction, student-content interaction, student-system interaction and teacher-system interaction. Yücel and Usluel (2016) point out that it is important to consider the type, quantity and quality of interactions. Each of these interactive behaviours is reflected in the logs. To facilitate their analysis, Moodle allows the extraction of these files in different formats: csv, xls, etc. The analysis of these files will provide a lot of information about the learning behaviours of the students, and the type of teaching of the professor. However, the information that can be obtained from Moodle log files is very extensive, so for a proper interpretation of the data it is necessary to use EDM (Agudo-Peregrina et al., 2014). Thus, EDM develops techniques and models that will facilitate the knowledge of students' learning behaviour patterns and the interactions between them (teacher-student, students-students). All of which supports continuous (formative) assessment processes. EDM can address different profiles (Romero and Ventura, 2007; Romero et al., 2013):

- Oriented towards students. This type of assessment is directed towards learning tasks and the aim is to improve the learning process in students.
- Oriented towards educators. The objective is to provide feedback to the teacher for instruction, evaluate the structure and contents of the course, analyse elements that have been effective in the learning processes, classify the type of students and see the needs for guidance and monitoring of learning. All this, to know the learning patterns of the students and the frequency of errors so that the teacher can implement the most effective activities.
- Oriented towards academics responsible and administration. The aim is to provide feedback to the institution to help improve learning platforms.

This functionality is very important in research on e-assessment models (Liyanage et al., 2016) that have to assess the learning strategies used by students, the environment in which learning takes place (Harrati et al., 2016), the design of teaching by the professor (Sáiz-Manzanares, 2018) and the use of active teaching methodologies (Sáiz-Manzanares & Montero-García, 2016), among others. In this area of research, it has been found that there are different patterns and learning outcomes depending on the type of e-assessment (Bogarín et al., 2018). In summary, the use of the methodologies described above will make it possible to detect patterns of student and teacher behaviour on the platform through the study of logs. Likewise, the EDMs will facilitate the study of behaviour and the development of cognitive-behavioural science (Jones, 2017).

### Detecting students at risk of failure through LMSs

**Fig. 1:**
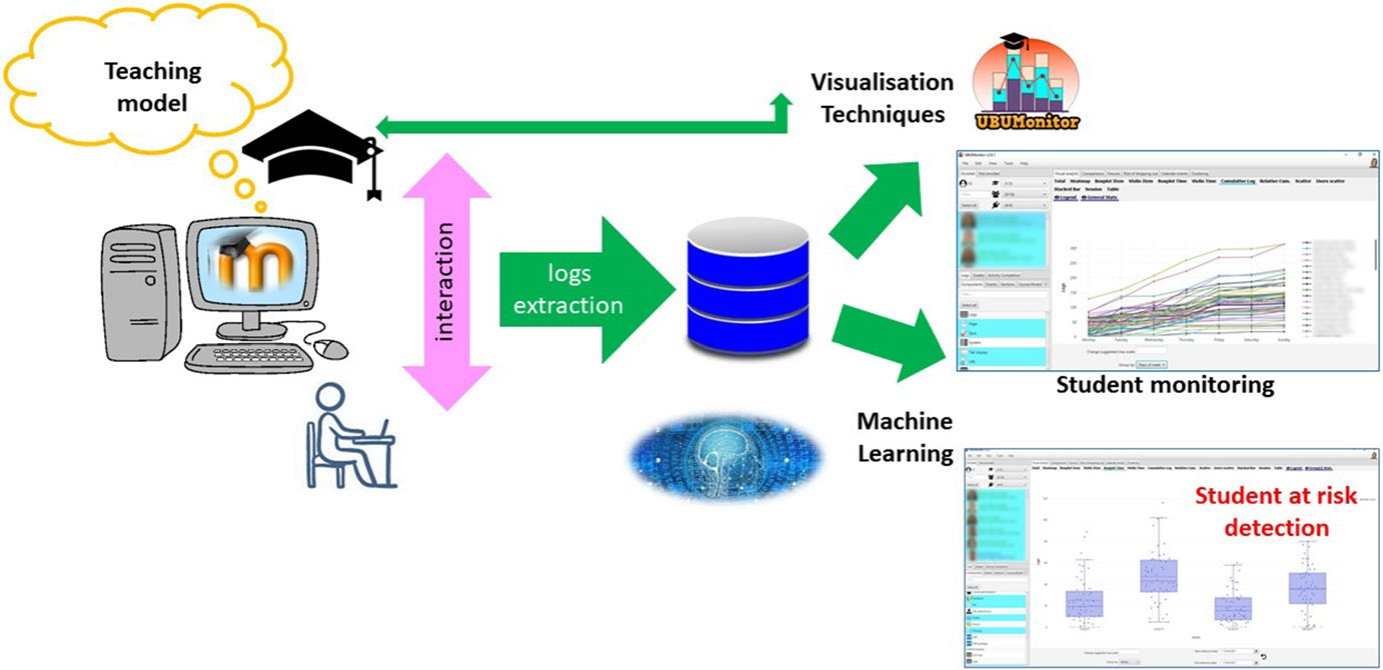
Diagram of the teaching–learning process in Moodle applying process monitoring tools

The detection of students at academic risk must be a priority objective for teachers and university institutions (Sáiz-Manzanares et al., 2017). In order to carry out an effective detection in LMSs, an analysis of the monitoring procedures that help to detect the learning patterns of each student is necessary (Cerezo, et al., 2017). These patterns explain up to 52% of the variance in learning outcomes. These studies are oriented from the use of EDM (Bogarín et al., 2018). In short, the frequency and systematicity of interaction on the platform by the learner together with the completion of self-assessment activities and average queries per day are aspects directly related to the achievement of effective learning (Sáiz-Manzanares et al., 2019b; Yücel & Usluel, 2016). The analysis of logs through EDM techniques will allow teachers to analyse the behaviour patterns of their students and predict the student at risk (Sáiz-Manzanares et al., 2019b). In addition, early intervention in these cases is likely to improve students' learning responses. Also, the use of LMSs will facilitate, especially in university settings, the structuring of collaborative teaching, which is expected to increase students' motivation towards the learning process (Järvelä et al., 2016). Recent studies confirm that monitoring students' learning patterns on the platform facilitates the discrimination of at-risk students with an explained variance of 67.2% (Bannert et al., 2014; Bogarín et al., 2018; Cerezo et al., 2016; Sáiz-Manzanares et al., 2021a). A summary of the points made in the introduction can be found in Fig. 1.

### Personalisation of learning and e-Guidance

Recent studies in Higher Education indicate that teaching methodologies should be directed towards more personalised forms of interaction with the student (Sáiz-Manzanares et al., 2019b). Therefore, Higher Education is in a moment of change derived from the new demands of the knowledge society. This fact has been increased by the pandemic situation due to COVID-19, which has led to teaching being increasingly carried out through LMSs (Sáiz-Manzanares et al., 2020b). The digitisation of teaching as mentioned above implies the inclusion of digital tools and teacher training programmes that address aspects especially related to the design of learning tasks, assessment methods and feedback to student learning outcomes (García-Peñalvo et al., 2020). For this reason, it is necessary for the university lecturer to carry out a guidance task in the learning process of each student. Thus, student guidance at university must be structured from the design of teaching programmes that promote successful learning (Carbonero et al., 2013). Understanding the guidance function as an inherent value of the teaching function (Sáiz-Manzanares & Román-Sánchez, 2011). In this field, new technologies have opened up a new environment for research in learning (Gros & García-Peñalvo, 2016; Lockee & Gros, 2020). As mentioned above, LMSs, such as Moodle, allow individualised monitoring of the teaching–learning process of each student. Recent studies indicate that personalised student tracking increases learning outcomes, frequency of interactions on the platform and motivation to learn. Likewise, such monitoring predicts students' learning outcomes by 61.3% and behaviour patterns in the LMS by 56.1% (Sáiz-Manzanares et al., 2017).

Based on the above-mentioned state of the art, the research questions of this work were:

#### Quantitative study

RQ1. Will there be significant differences between students' behaviours in the LMS as a function of the implemented teaching modality influenced by the covariate collaborative group?

RQ2. Will there be significant differences between students’ learning outcomes in the LMS as a function of the implemented teaching modality influenced by the covariate collaborative group?

RQ3. Are there significant differences in students' satisfaction with the development of the teaching–learning process in the LMS depending on the implemented teaching modality influenced by the covariate collaborative group?

RQ4. Will the grouping clusters with respect to interactions in the LMS match the categorisation of collaborative groups with respect to achievement of learning outcomes?

#### Qualitative study

RQ5. Do students in each collaborative group have different behavioural patterns in each teaching modality?

**Fig. 2:**
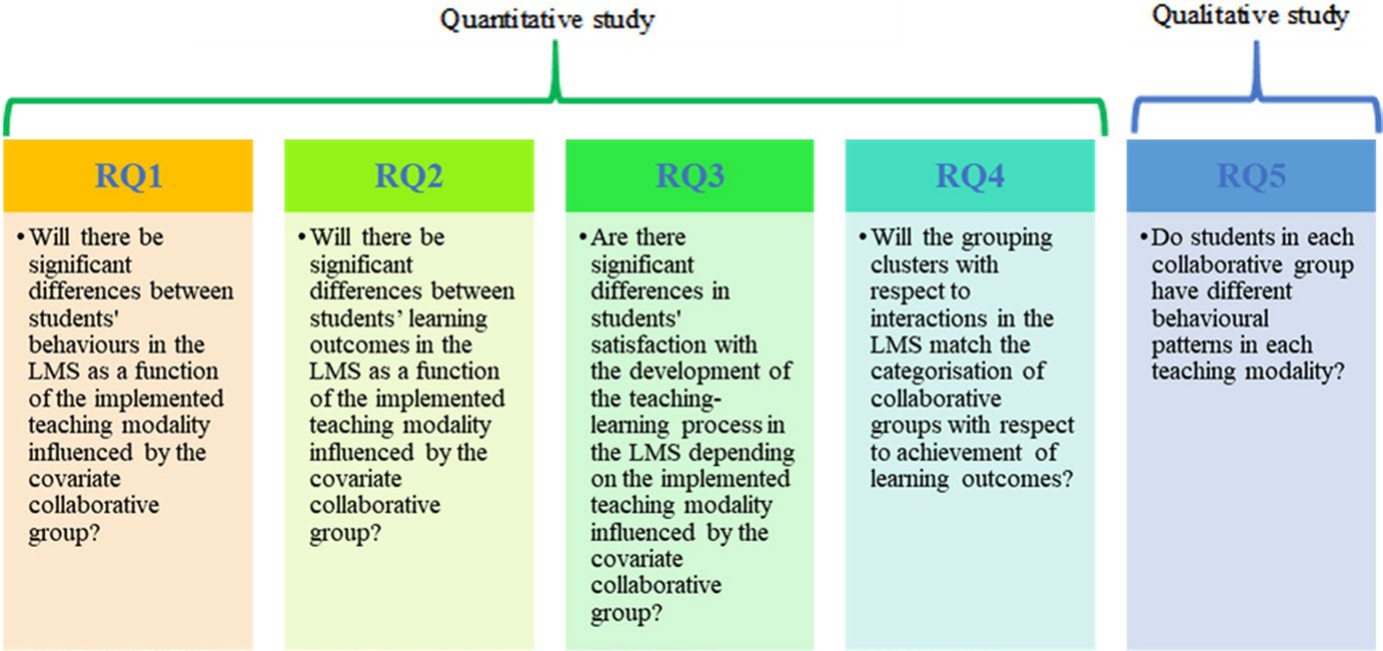
Outline of relationship between type of study and research questions

A diagram of the procedure followed in each study can be found in Fig. 2.

## Method

### Participants

We worked with a sample of 143 students of third year in the Occupational Therapy degree applying convenience sampling, which was divided into three groups of the same subject. In each of them, a type of teaching modality was implemented (see the procedure section), three in total. In Mode A there were 55 students (49 women, mean age (Mage) = 22.6 and standard deviation (SDage) = 3.5 and 6 men, Mage = 23.7 and SDage = 1. 9), 42 students in Mode B (34 women Mage = 22.3; SDage = 1.6, and 8 men Mage = 22.5, SDage = 2), 46 students in Mode C (38 women, Mage = 22.4; SD = 2.25 and 8 men, Mage = 21.6; SD = 1.8). The higher percentage of women than men is common in Health Sciences, where the ratio according to the latest report of the Spanish University Rectors' Conference -CRUE- (Hernández-Armenteros and Pérez-García, 2019) is 73.8, an aspect that is confirmed in this study.

Within each modality the participants were in collaborative groups (they could choose with whom to form the group) made up of 3 to 6 participants, exceptionally and due to personal reasons of the students there could be groups made up of only one student, these groups were eliminated in this study. In Modality A, 13 groups were registered, in Modality B, 12 groups and in Modality C, 11 groups.

### Instruments

**Fig. 3:**
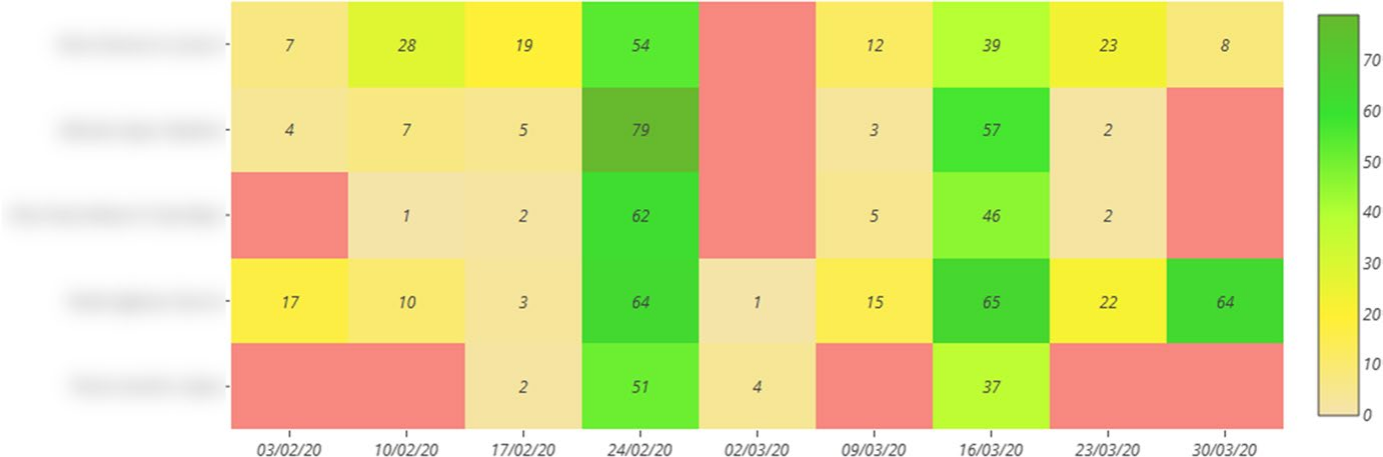
Heat map of the weekly monitoring of each of the students in a collaborative group in different components in Moodle

- UBUVirtual Platform. This platform is an LMS developed in a Moodle environment, version 3.9 was used.
- Learning strategies scale (ACRA) by Román-Sánchez and Gallego Rico (2008). This scale is a highly contrasted instrument in research on learning strategies in Spanish-speaking populations (Carbonero et al., 2013). ACRA identifies 32 strategies at different stages of information processing. In this study, only the metacognitive scales were used, which include the subscales of self-knowledge, self-planning, and self-evaluation. It also has a Cronbach's reliability coefficient of α = 0.90, an inter-rater construct validity of r = 0.88 and a content validity of r = 0.88.
- Design of the subject. A teaching methodology based on the use of OPBL was applied. Especifically, the course included five thematic units with the following structure: presentation of the unit, additional information, quiz-type questionnaires on the platform with automatic feedback and a satisfaction survey in each unit. However, dissimilar elements were differentiated depending on the teaching intervention modality: Modality A, included PBL, automatic product-oriented feedback on the answers given by the student (students were given information on the results obtained in the different activities) and Flipped Classroom experiences; Modality B, included PBL, automatic process-oriented feedback (students were given information on the results obtained in the different activities and information was included on why each answer was given and where they could find this information on the platform), Flipped Classroom experiences and virtual laboratories; Modality C, included PBL methodology, product-oriented feedback, Flipped Classroom experiences, virtual laboratories and the help of an Intelligent Personal Assistant (IPA) which informed the student about the events of the subject (Sáiz-Manzanares et al., 2020a).
- Learning outcomes. The following assessment procedures were considered: PBL elaboration (had a weight of 25% in the total grade), PBL Presentation (had 20% of the final weight), Quiz (had 30% of the final weight). For this study, the grades in the practical exercises (25% of the weight) were not considered, as all students obtained the same grade (the methodology of "learning from error" was used, when the practical exercise did not obtain the maximum grade, it was returned for improvement until the student obtained the maximum grade, so this score was not discriminating), the total of the Learning outcomes was 100%.
- UBUMonitor tool (Ji et al., 2018). UBUMonitor is a desktop application running on the client, implemented with Java, and with a graphical interface developed in JavaFX. The application connects to the selected Moodle server, through web services and REST API provided by the server. In the absence of web services to retrieve specific data, web scraping techniques are also used. All communication between the Moodle server and the UBUMonitor client is encrypted via HTTPS for security reasons. As a result of these queries, the data is obtained in JSON and CSV format, processed and transformed into Java objects in the client. For the visualisation of the collected data, the hybrid solution of applying Java and embedding web pages with different graphical JavaScript libraries within the desktop application is used. The data can be stored on the client to optimise access times for later queries and offline access to the data, using the serialisation mechanism available in Java. The serialised files with the subject data are stored encrypted with the Blowfish algorithm (Schneier, 1993). The application, which is open source and free of charge, includes six modules: visualisation (which offers the representation of the frequencies in different graphs: Heat Map, Boxplot, Violin, Scatter, etc.), comparison, forums, risk of dropping out (allows the detection of students who have not been connected for 7–15 days at certain times during the course), Calendar events and Clustering (allows for finding clusters by applying different algorithms such as *k*-means, Fuzzy *k*-means, etc.). Especifically, in this study we have used the visualisation module, which allows an analysis of the access frequencies in components, events, sections or course seen in Moodle with options to analyse the logs in different graphs (Heat Map has been chosen, as it offers the results with a numerical and colour visualisation of intensity throughout the duration of the course) in the development of the subject. All visualisation options allow export in graphical format and in.csv format, for the elaboration of reports and their subsequent analysis with other tools. The use of visualisation techniques such as Heat Map for the detection of students at risk is a tool that is proving to be very effective (Dobashi et al., 2019; Sáiz-Manzanares, et al., 2020b). An example of student monitoring within a collaborative group can be found in Fig. 3.

### Procedure

We worked with three groups of third-year students in the Occupational Therapy Degree at the Faculty of Health Sciences of the University of Burgos. Prior to the study, a positive report was obtained from the bioethics committee of the University of Burgos, followed by written informed consent from all study participants. Before the intervention, the normality of the sample distribution and the homogeneity of the groups in the results of the Metacognitive Strategies Scale of Román-Sánchez and Gallego Rico (2008) were checked. This scale was applied to each group of students within each teaching intervention modality. Table 1 shows an outline of the teaching methodologies in each intervention modality applied. The distribution of the teaching methodology was carried out using convenience sampling. The teaching was given by the same professor in order to eliminate the extraneous variable "type of teacher".

**Table 1:** Modalities of intervention with their corresponding teaching methodologies applied

Modality	Teaching methodology
Modality A	Online Project-based learning (OPBL) Quizzes with product-oriented feedback Flipped Classroom
Modality B	Online Project-based learning (OPBL) Quizzes with process-oriented feedback Flipped Classroom Virtual laboratories
Modality C	Online Project-based learning (OPBL) Quizzes with process-oriented feedback Flipped classroom Virtual laboratories Intelligent Personal Assistant (IPA)

**Fig. 4:**
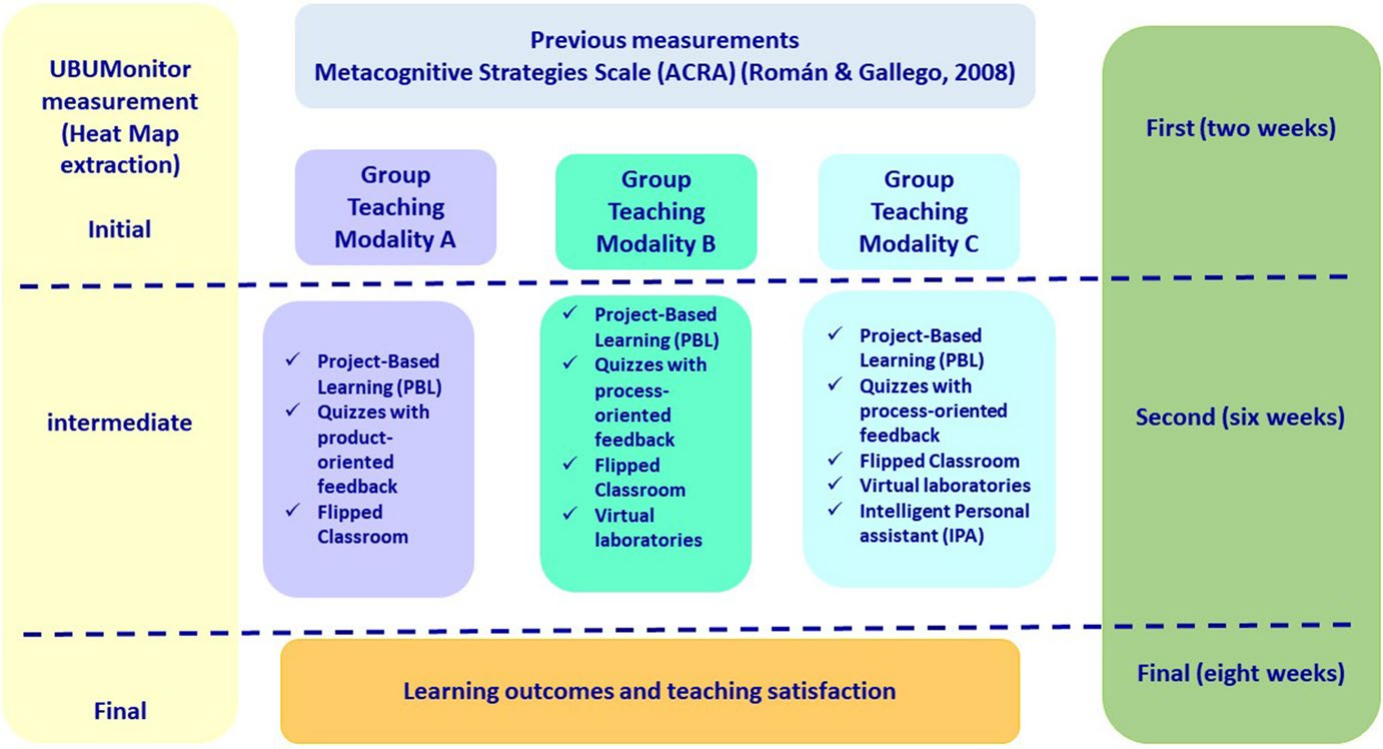
Research development procedure

The students' learning behaviour was studied in Moodle throughout the course in the three teaching modalities. In all the modalities, students worked with the OPBL methodology and were distributed in collaborative groups that were formed according to students' preferences, the ratio of the groups ranging between 3 and 6 members. Also, the learning outcomes in the different assessment procedures were analysed. The performance of each collaborative group was categorised with respect to the total grade in three categories: (1) medium performance: scores between 7.9 and 8.5; (2) high performance: scores between 8.5 and 9.5 and (3) very high performance: scores between 9.6 and 10), the category of low performance was not applied, as the lowest score obtained by the students in Learning outcomes total was equal to 7.9. The duration of the teaching in the three modalities was 9 weeks, and follow-up measurements were established with the UBUMonitor tool in which the Heat Maps were found: a first initial follow-up measurement after two weeks, an intermediate measurement after six weeks and a final measurement after eight weeks. A summary of the procedure is presented in Fig. 4.

### Designs

Both quantitative and qualitative research designs were applied to test the research questions. For the quantitative research a 3 × 5 × 4 factorial quasi-experimental design was used (Campbell and Stanley, 2005) and for the qualitative research (Anguera et al., 2018) a longitudinal comparative design was used (Flick, 2014).

### Data analysis

An analysis of the normality of the sample was carried out by applying skewness and kurtosis statistics, then to check the homogeneity in the three groups before the intervention in the results of the Metacognitive Strategies Scale (Román-Sánchez & Gallego Rico, 2008) a one-factor ANOVA was used with fixed effects "modality type" and eta-squared effect value (η^2^) [a small effect is considered to be the interval between 0.10 and 0.29, a medium effect the interval between 0.30 and 0.49 and high the interval between 0.50 and 1 (Cohen, 1988)]. Subsequently, to test Research Questions (RQ) RQ1, RQ2 and RQ3, a one-factor ANCOVA with fixed effects "modality type" and covariate "collaborative group" was applied. To contrast RQ4, Principal Component Analysis (PCA), Machine Learning techniques of unsupervised learning (clustering), ANOVA, cross-tabulation, and Pearson's contingency coefficient (this expresses the intensity of the relationship between two or more qualitative variables. It is based on the comparison of the actually calculated frequencies of two characteristics with the frequencies that would have been expected irrespective of these characteristics) are used. The statistical package SPSS v.24 (IBM, 2016) was used to perform these analyses. In addition, visualisation techniques, in particular Heat Map, were used to contrast RQ5 using the UBUMonitor software (Ji et al., 2018). Also, qualitative analysis techniques of Heat Map categorisation and analysis of the frequencies found in the categorisation and Sankey plots were used (this is a specific flow chart in which the width of the bands is proportional to the amount of flow and serves to visualise transfers of X elements between processes. This type of diagram puts a visual emphasis on the important transfers within a system and helps to locate the dominant contributions to a total flow). The software ATLAS.ti 9 (ATLAS.ti, 2020) was used for its generation.

## Results

### Previous statistical analyses

Previously, in order to check the normality of the distribution, the skewness and kurtosis statistics were found with respect to the results on the Metacognitive Strategies Scale of Román-Sánchez and Gallego Rico (2008). No extreme values were found for skewness [Self-knowledge A = −1.18; Self-planning and regulation A = −0.36; Self-assessment A = −1.06, extreme values are considered those greater than |2.00|] or kurtosis [Self-knowledge K = 3.49; Self-planning and regulation K = 0.53; Self-assessment K = 2.71, extreme values are considered those between |8.00| and |20.00|] (Bandalos & Finney, 2001). (Bandalos & Finney, 2001) so it can be deduced that the sample follows a normal distribution. Therefore, parametric statistics were applied.

Next, in order to check the homogeneity between the groups before the intervention in the three modalities, a one-factor ANOVA with fixed effects " modality type" was carried out with respect to the results found in the Román-Sánchez and Gallego Rico (2008) metacognitive strategies scale. As can be seen in Table 2, no significant differences were found between the students assigned to the three modalities. Thus, parametric statistics were applied to test the research questions within the quantitative research study.

**Table 2:** ANOVA of a fixed effects factor "modality type" in the ACRA Metacognitive Strategies scale (Román-Sánchez & Gallego Rico, 2008)

Metacognitive scale	N	*n*	G1	*n*	G2	*n*	G3	*df*	*F*	*p*	η^2^
			*M (SD)*		*M (SD)*		*M (SD)*				
Self-knowledge	143	55	20.6 (2)	42	20.2 (2.5)	46	19.6 (3.2)	(142,2)	1.7	0.2	0.02
Self-planning	143	55	11.8 (2.5)	42	11.9 (2.5)	46	12.2 (2.4)	(142,2)	0.4	0.6	0.006
Self-assessment	143	55	19.24 (2.7)	42	18.7 (2.8)	46	19.3 (3.3)	(142,2)	0.5	0.6	0.006

G1 = Modality A; G2 = Modality B; G3 = Modality C; *M* = Mean; *SD* = Standard Deviation; *df* = degrees of freedom, η^2^ = eta-squared effect value

### Quantitative study

To test RQ1, a one-factor fixed-effect ANCOVA was performed on the type of modality and the covariate collaborative group. Significant differences were found in all types of accesses as well as in the average number of visits per day between the three modalities. However, there was no significant effect of the covariate "collaborative group". The highest averages for accesses to complementary information, accesses to the guidelines for OPBL, as well as the average number of visits per day were found in Modality C. Nevertheless, in Modality A, the highest mean was found for accesses to feedback. The effect values were medium for accesses to Supplementary Information and mean number of visits per day and low for the remaining variables (see Table 3).

**Table 3:** ANCOVA of a fixed effects factor "modality type", covariate "collaborative group" with respect to platform accesses

Type of access	N	*n*	G1	*n*	G2	*n*	G3	*df*	*F*	*p*	η^2^
			*M (SD)*		*M (SD)*		*M (SD)*				
*Independent variable*											
Supplementary information	143	55	38.02 (23.45)	42	19.36 (13.23)	46	77.91 (45.12)	(142, 2)	41.00	0.00*	0.37
OPBL Guidelines	143	55	5.09 (6.30)	42	11.86 (8.24)	46	31.22 (29.35)	(142, 2)	26.72	0.00*	0.28
Co-evaluation	143	55	26.93 (16.17)	42	7.93 (6.76)	46	22.15 (17.70)	(142, 2)	20.71	0.00*	0.23
Feedback	143	55	116.78 (45.84)	42	71.71 (24.40)	46	78.91 (25.04)	(142, 2)	24.53	0.00*	0.26
Average number of accesses per day	143	55	3.54 (1.21)	42	1.90 (0.51)	46	4.49 (1.68)	(142, 2)	40.69	0.00*	0.40
*Co-variable*											
Supplementary information	143	55		42		46		(142, 2)	0.17	0.68	0.001
OPBL Guidelines	143	55		42		46		(142, 2)	0.18	0.90	0.000
Co-evaluation	143	55		42		46		(142, 2)	0.005	0.95	0.001
Feedback	143	55		42		46		(142, 2)	0.13	0.72	0.001
Average number of accesses per day	143	55		42		46		(142, 2)	0.15	0.70	0.001

^*^p < 0.05. Note: G1 = Modality A; G2 = Modality B; G3 = Modality C; *M* = Mean; *SD* = Standard Deviation; *df* = degrees of freedom, η^2^ = eta squared effect value

To test RQ2, a one-factor ANCOVA with fixed effects was performed on the type of modality and the covariate collaborative group. Significant differences in learning outcomes were found in the assessment procedures PBL elaboration, PBL presentation and in total learning outcomes and were not found in quizzes. Also, the effect values were low in all learning outcomes except PBL elaboration and Learning outcomes total, which were medium. In addition, the effect of the covariate "collaborative group" was found in all types of learning outcomes (see Table 4).

**Table 4:** ANCOVA of one fixed effects factor "modality type", covariate "collaborative group" on learning outcomes

Type of access	N	*n*	G1	*n*	G2		G3	*df*	*F*	*p*	η^2^
			*M (SD)*		*M (SD)*		*M (SD)*				
*Independent variable*											
PBL elaboration			2.29 (0.12)	42	2.35 (0.10)	46	2.19 (0.19)	(142, 2)	13.93	0.00*	0.17
PBL Presentation			1.70 (1.17)	42	1.66 (0.24)	46	1.90 (0.13)	(142, 2)	36.74	0.00*	0.35
Quiz			2.72 (0.24)	42	2.63 (0.13)	46	2.65 (0.22)	(142, 2)	2.21	0.11	0.03
Learning outcomes total			9.09 (0.49)	42	8.90 (0.40)	46	8.90 (0.47)	(142, 2)	2.50	0.09	0.04
*Co-variable*											
PBL elaboration				42		46		(142, 2)	61.85	0.00*	0.31
PBL Presentation				42		46		(142, 2)	33.34	0.00*	0.19
Quiz				42		46		(142, 2)	31.49	0.00*	0.19
Learning outcomes total				42		46		(142, 2)	114.85	0.00*	0.45

G1 = Modality A; G2 = Modality B; G3 = Modality C; *M* = Mean; *SD* = Standard Deviation; *df* = degrees of freedom, η^2^ = eta-squared effect value

^*^p < 0.05

Likewise, the drop-out rate in modality A and B was 0% and in modality C 0.02%, and the success rates (percentage of successful students out of the students presented in the first and second sittings) were 98.2% in Modality A; 100% in Modality B; and 100% in Modality C, respectively. This is relevant considering that the average success rate in the other subjects of the academic year was, respectively, 91.9%, 71.2% and 83.9%. This indicates a difference of 6.3, 28.8 and 16.1 percentage points.

To test RQ3, a one-factor fixed-effects ANCOVA was performed on the type of modality and the covariate collaborative group. No significant differences were found in student satisfaction with the teaching modality (see Table 5).

**Table 5:** ANCOVA of one fixed effects factor "modality type”, covariate "collaborative group" on student satisfaction with teaching

Type of access	N	*n*	G1	*n*	G2		G3	*df*	*F*	*p*	η^2^
			*M (SD)*		*M (SD)*		*M (SD)*				
*Independent variable*											
Satisfaction with teaching			4.40 (0.39)	42	4.38 (0.33)	46	4.48 (0.32)	(141, 2)	1.30	0.28	0.002
*Co-variable*											
Collaborative group									1.30	0.28	0.02

G1 = Modality A; G2 = Modality B; G3 = Modality C; *M* = Mean; *SD* = Standard Deviation; *df* = degrees of freedom, η^2^ = eta-squared effect value

^*^p < 0.05

To test RQ4 beforehand, a Principal Component Analysis (PCA) was performed and a KMO = 0.22, χ^2^ = 124.87, *p* = 0.00 was obtained. Two components were isolated: component 1, which included the following dependent variables: accesses to complementary information, explained variance = 0.83; accesses to co-evaluation, explained variance = 0.84 and average number of views per day, explained variance = 0.69 and component 2, which included accesses to feedback, explained variance = 0.98 and accesses to guidance to perform the PBL, explained variance = 0.65. Both components explained 68.50% of the variance.

Next, a cluster analysis was carried out using the *k-means* algorithm with respect to the students' access to the platform in the three teaching modalities. Three grouping clusters were found. Cluster 2 was considered as: Excellent cluster 2, as it had the best values for most of the attributes, except for accesses to teacher feedback which was ranked second; Good, cluster 1, and Acceptable cluster 3. None of the clusters were considered to have bad values, as the data reflected interaction in the LMS for all types of accesses (see Table 6).

**Table 6:** Final cluster centres

Access	Cluster		
	Acceptable n = 69	Good n = 50	Excellent n = 24
Supplementary information	28	35	116
OPBL Guidelines	13	5	44
Co-evaluation	10	25	35
Feedback	64	130	91
Average visits per day	2.26	3.67	5.87

Also, an ANOVA was performed between the values found in the clusters between all types of accesses (see Table 7).

**Table 7:** ANOVA between clusters

Type of access	N	*Cluster mean square*	*Root mean square error*	*df*	*F*	*p*
Supplementary information	143	71963.80	461.73	(2,140)	155.86	0.0001*
OPBL Guidelines	143	12552.26	260.38	(2,140)	48.21	0.0001*
Co-evaluation	143	6722.31	182.97	(2,140)	36.74	0.0001*
Feedback	143	62796.68	709.24	(2,140)	88.54	0.0001*
Average visits per day	143	119.63	1.14	(2,140)	104.62	0.0001*

*p < 0.05

Next, we tested whether the variables selected as indicators of LMS use were equally sustainable in the cluster configuration. The three clusters explained 66% variance [Wilks' Lambda = 0.12; *F* (5, 10) = 52.68; *p* < 0.000, η^2^ = 0.66]. This implies that students had different behavioural patterns of learning in the three clusters across the five types of access. However, not all access types had the same degree of discrimination. In the analysis of intergroup differentiation the variables that contributed most to differentiation were: accesses to supplementary information with a high effect value [*F*(2, 140) = 155.86, *p* < 0.000, η^2^ = 0.70]; mean number of visits per day [*F*(2*,* 140) = 104.62, *p* = 0.000, η^2^ = 0.60], the accesses to the feedback given by the teacher with a mean effect value [*F*(2, 140) = 88.54, *p* = 0.000, η^2^ = 0.56], the accesses to the OPBL orientations with a mean effect value [*F*(2, 140) = 48.21, *p* = 000, η^2^ = 0.41] and the accesses to the co-assessment with a mean effect value [*F*(2, 140) = 36.74, *p* = 0.000, η^2^ = 0.34].

Finally, the relationship between the distribution of the clusters and the categorisation of the learning outcomes in the collaborative groups was cross tabulated and the contingency coefficient was found to be *C* = 0.24, not significant at 95% *p* = 0.06 (see Table 8). This shows that the relationship between the assignment clusters and the categorisation of the collaborative groups was small. In other words, the grouping of students in the clusters does not exactly match the categorisation of students' performance according to the learning outcomes obtained in each of the collaborative groups. This fact may be an indicator that students' behaviour in Moodle is not homogeneous within each of the collaborative groups.

**Table 8:** Crosstabulation: number of cluster cases by cluster categorisation with respect to the collaborative group

		Categorisation of Learning outcomes in collaborative groups						*n*
		1 *n* = 28	%	2 *n* = 94	%	3 *n* = 21	%	
Cluster case number	1	10	20	27	54	13	26	50
	2	5	41.67	18	75	1	4.16	24
	3	13	18.84	49	70.01	7	10.50	69

Categorisation of Learning outcomes in the collaborative groups 1 = medium performance: scores between 7.9 and 8.5, 2 = high performance: scores between 8.5 and 9.5 and, and 3 = very high performance: scores between 9.6 and 10.

### Qualitative study

**Fig. 5:**
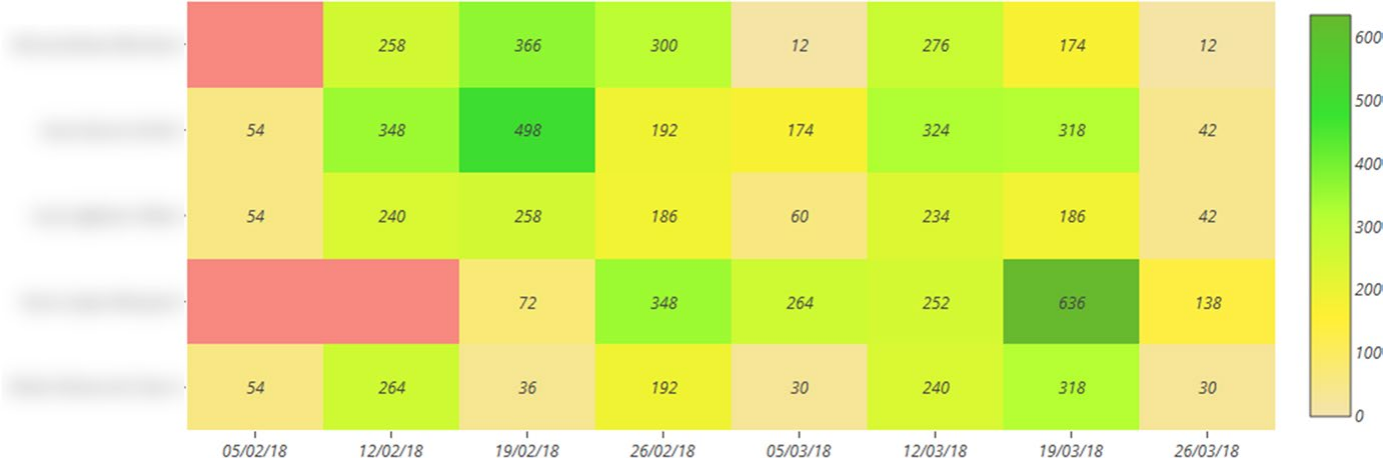
Heat map on the weekly monitoring of a collaborative group in Moodle

In order to test RQ5, a qualitative analysis was carried out in which the following steps were applied:

*Step 1* Heat maps were found in each of the collaborative groups within each teaching modality using the UBUMonitor tool (Ji et al., 2018, 2020). An example of the Heat Maps found can be found in Fig. 5.

*Step 2* Heat Map images from each collaborative group were included in the software for the Atlas.ti 9 qualitative data analysis software.

*Step 3* Heat Map were categorised in each collaborative group by teaching modality, establishing the following criteria according to the frequency of observed non-interaction: “Continuous work throughout the subject” (implies continuous student work throughout the subject, i.e. frequency of access in all weeks); “Non-interaction at the start of the subject” (implies non-interaction in the first two weeks of the subject); “Non-interaction in the middle of the subject” (implies non-interaction between the third and sixth week) and “Non-interaction at the end of the subject” (refers to non-interaction between the seventh and eighth week).

*Step 4* Sankey plots were found for each modality. Also, a frequency analysis was carried out for each of the categorisation criteria established in each teaching modality.

**Fig. 6:**
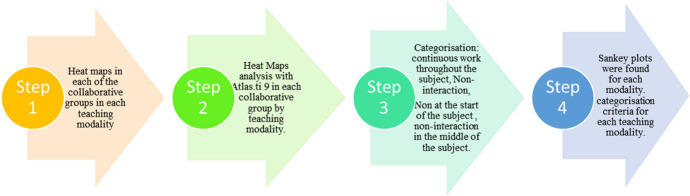
Steps followed in the qualitative study

A schematic of the steps followed in the qualitative study is presented in Fig. 6.

**Fig. 7:**
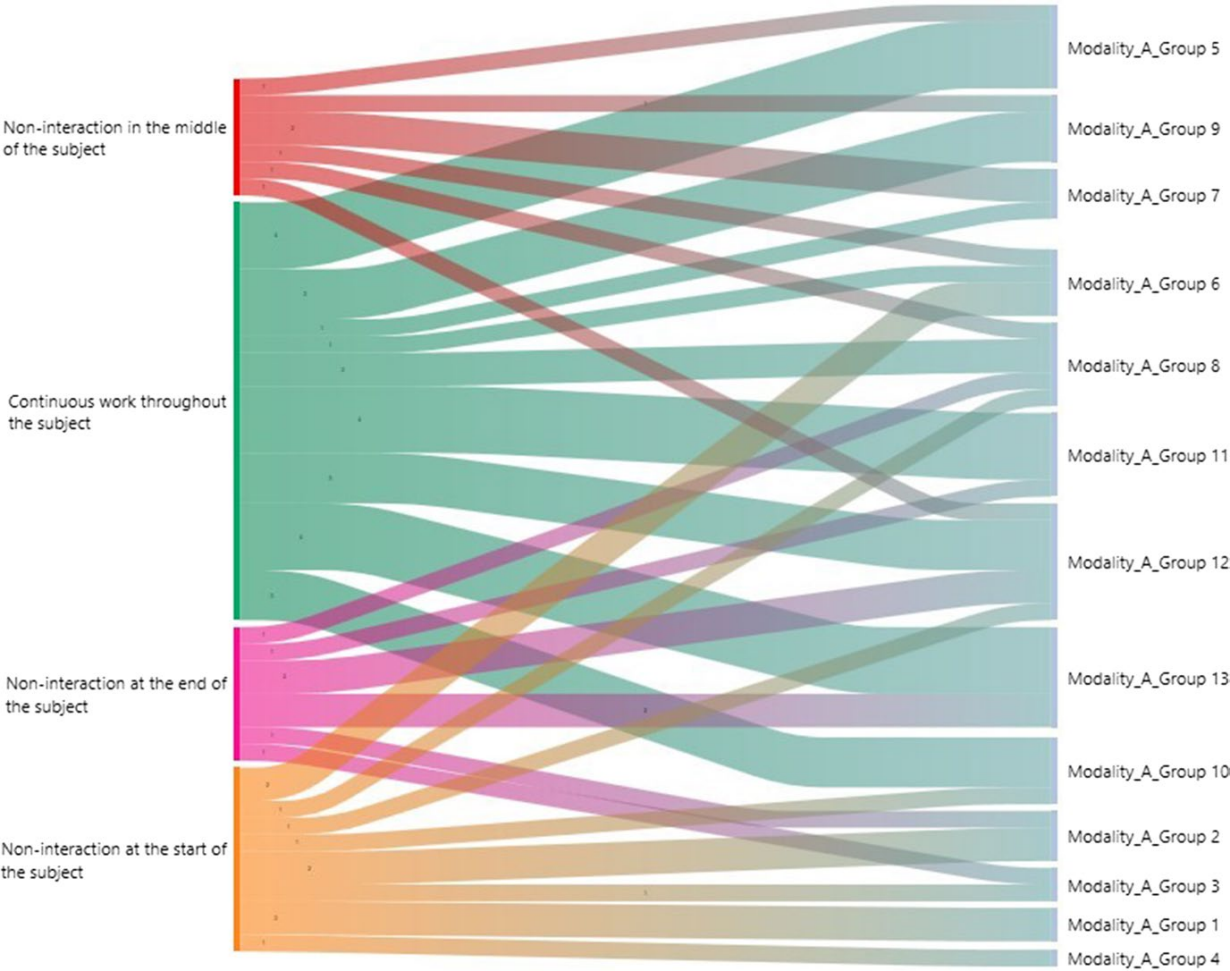
Sankey chart in teaching Modality A

Figure 7 shows the analysis of frequencies per categorisation criterion in a Sankey chart within Modality A. The groups with the highest frequencies (≥ 50%) for the criterion "Continuous work throughout the subject" were groups 5, 9, 10, 11 and 13. The group with the lowest interactions (≥ than 50% non-interaction) with “Non-interaction at the end of the subject” was group 3. The groups with the lowest interactions (≥ 50% non-interaction) at the start of the subject were 1, 2, 3, 4, and 6. The group with the lowest interactions (≥ 50% non-interaction) in the middle of the subject was group 7.

**Fig. 8:**
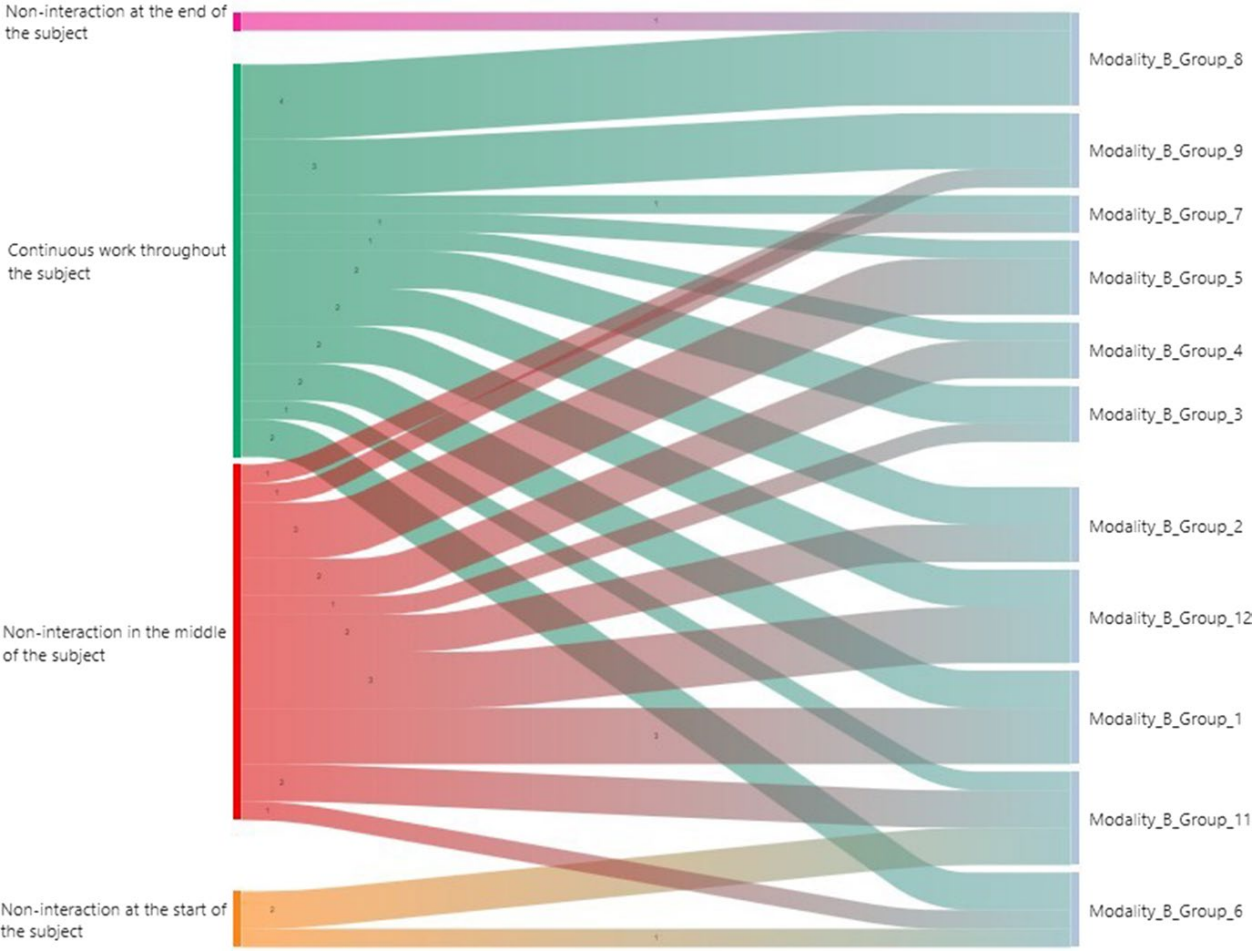
Sankey chart in teaching Modality B

Figure 8 shows the distribution of the collaborative groups within Modality B: the groups that worked continuously (≥ 50%) were collaborative groups 3, 6, 7, 8, and 9. The groups with a lower interaction (≥ 50%) towards the middle of the course were groups 1, 2, 4, 5, 7, and 12. Likewise, no non-interactions (≥ 50%) were detected at the start or end of the subject.

**Fig. 9:**
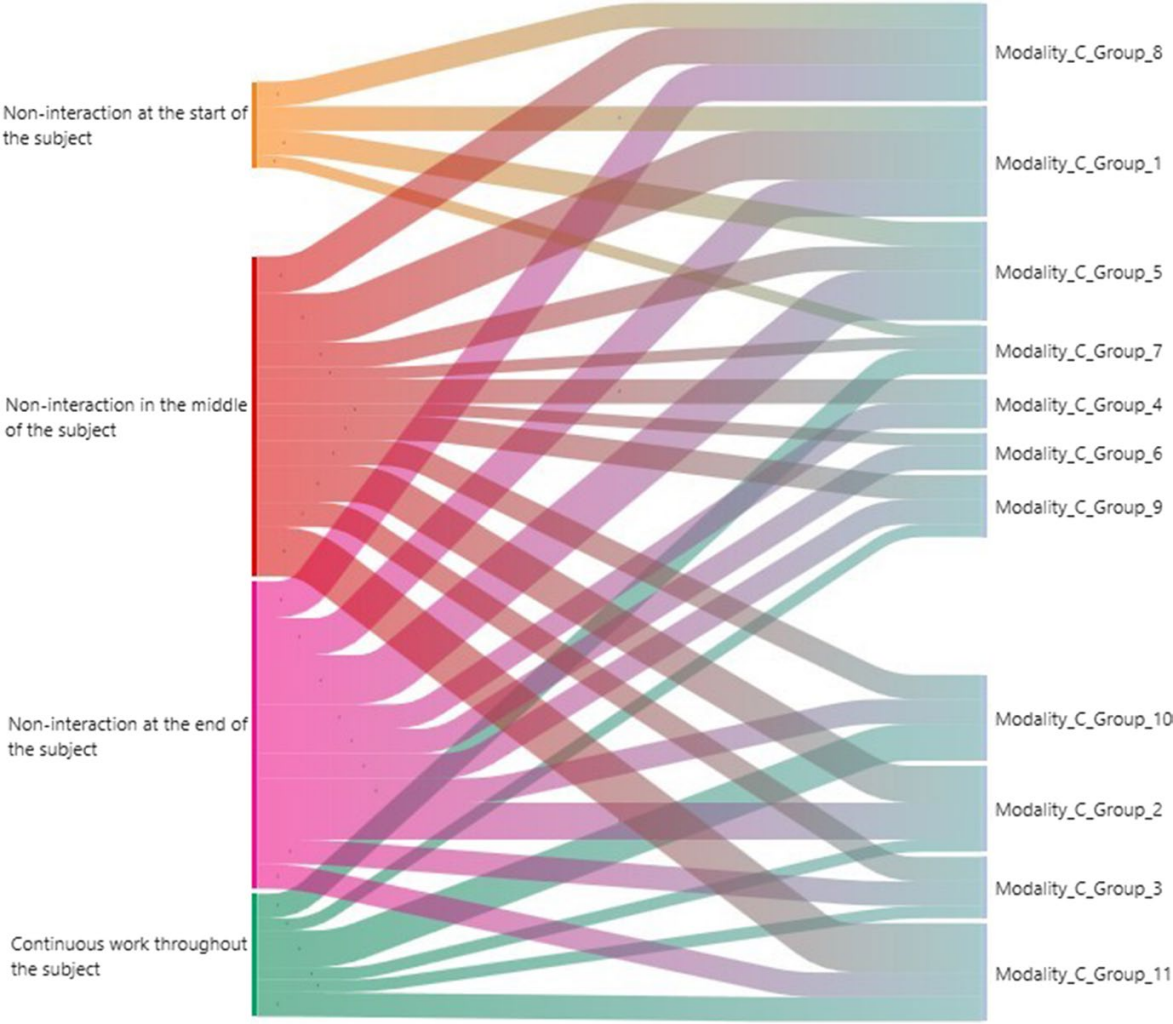
Sankey chart in teaching Modality C

Figure 9 shows the distribution of the collaborative groups within Modality C: the collaborative groups that had continuous interaction (≥ 50%) throughout the course in this case was group 7. The collaborative groups in which non-interaction (≥ 50% non-interaction) was detected towards the middle of the course were groups 4 and 11, and at the end of the course groups 4, 5, and 6. Likewise, no non-interactions (≥ 50%) were detected at the start of the subject.

## Discussion

Regarding RQ1 (Will there be significant differences between students' behaviours in the LMS as a function of the implemented teaching modality influenced by the covariate collaborative group?), it was found that the teaching design does influence student behaviour in Moodle, although no effect of the covariate "collaborative group" was found. Specifically, the average number of accesses was higher in Modality C in the additional information, the guidelines for taking the OPBL and the average number of visits per day. However, a higher rate of accesses to the feedback was found in Modality A. The explanation may be that in Modality C a process-oriented feedback was used in which the score obtained was explained to the students with a detailed explanation of the reason for each correct or incorrect answer in addition to the grade while, in Modality A, a product-oriented feedback was used, in this case only information about the grade was given to the students. Concerning RQ2 (Will there be significant differences between students’ learning outcomes in the LMS as a function of the implemented teaching modality influenced by the covariate collaborative group?), the type of teaching modality was found to influence learning outcomes in all assessment tests except for quiz-type tests. In this case, the covariate collaborative group type did have an effect on all learning outcomes. The explanation may be that quiz grades involve individual rather than collaborative work. Therefore, the group does not compensate for the results, and in the other assessment tests collaborative work improves the learning outcomes of each student. Thus, it can be concluded that the teaching design seems to directly affect the collaborative work of the groups (Sáiz-Manzanares, 2018). Related to this explanation is the hypothesis that one of the members of the collaborative group performs leader functions and makes the differences within the members of each group compensate (Järvelä et al., 2016). In this line, the results found in the qualitative analysis corroborate this hypothesis as differences in behaviour between each of the members in Moodle have been found and visualised in the heat maps. These results verify the findings of Park and Jo (2017) and Yilmaz et al. (2020). In summary, each student has a learning style (Harrati et al., 2016) and this is an element to be considered by the teacher for the design of the subject (Sáiz-Manzanares et al., 2021a). Based on this, the learning environment in the LMS has to offer students several resources (visual, auditory and blended) and different assessment procedures (Bogarín et al., 2018) including process-oriented feedback and not only product-oriented feedback (Aikina & Bolsunovskaya, 2020; Chen et al., 2020; Liyanage et al., 2016) so that each learner can choose to access information in the way that best relates to the method they learn (De Kock, 2016; Sáiz-Manzanares et al., 2019c).

Regarding RQ 3 (Are there significant differences in students' satisfaction with the development of the teaching–learning process in the LMS depending on the implemented teaching modality influenced by the covariate collaborative group?) no significant differences were found between the three modalities implemented. One possible explanation is that all the teaching modalities studied included a constructivist methodology based on OPBL (Chen et al., 2020; Sáiz-Manzanares & Montero-García, 2016) with Flipped Classroom experiences (Yilmaz et al., 2020) and feedback to the assignments implemented in Moodle. Such teaching modality facilitates SRL and motivation towards learning (Järvelä et al., 2016; Noroozi et al., 2019).

With regard to RQ4 (Will the grouping clusters with respect to interactions in the LMS match the categorisation of collaborative groups with respect to achievement of learning outcomes?) this study aimed to find out was whether the grouping carried out in terms of the categorisation of the collaborative groups (very good, good, acceptable) with respect to the learning outcomes in Moodle corresponded with the grouping that could be obtained through clustering techniques, and it was found that there was no correspondence. This fact reinforces the idea that the behaviour of the members of a group is not homogeneous and that within each group there is a leader (Yilmaz et al., 2020). It has been shown that this type of dynamic within the collaborative group is more effective for the overall group performance than a structure on the same level as suggested by Doise et al. (1975).

Finally, concerning RQ5 (Do students in each collaborative group have different behavioural patterns in each teaching modality?) it has been found that the behavioural profiles within each collaborative group, that are represented in Heat Map, do not have a homogeneous pattern of interaction between the members of each collaborative group and that there are always one or two members in each group who set the pace of work (Dobashi et al., 2019; Sáiz-Manzanares et al., 2020b). Therefore, it can be concluded that monitoring the learning process in each student is essential throughout the entire development for the detection of students at risk, especially in the initial and intermediate phases of the learning process (Bannert et al., 2014; Bogarín et al., 2018; Cerezo et al., 2016; Sáiz-Manzanares et al., 2021b). Ideally, an initial measurement should be taken two weeks into the course, an intermediate measurement (in the middle of the course) and a final measurement (one or two weeks before the end of the course). Also, different interaction patterns have been found within each of the collaborative groups in each teaching modality among the members of each workgroup. In order to know this data, the LMS needs to have this analysis functionality or enable connection to tools such as UBUMonitor (Ji et al., 2018) that allow the visualisation of this data to be easily consulted using different techniques (Heat Map, Boxplot, Scatter, Stacked Bar, etc.). UBUMonitor has proved to be a very useful tool for this purpose, as it allows for easy monitoring of each of the students in Moodle at different periods of the teaching process (analysis by days, weeks or months) in the different components. In addition, this resource facilitates more complete EDM studies such as cluster analysis (Agudo-Peregrina et al., 2014).

In sum, the qualitative micro-analysis of the behaviour of small groups in LMSs applying OBPL is diverse, although a more or less systematic interaction can be detected throughout the course of the subject. It is relevant that despite the differences, no non-interaction was detected in any of the modalities during the course. There are, however, intra- and inter-modal oscillations. Therefore, future studies will be aimed at finding out which are the best interaction patterns and which differentiating characteristics (students' motivation, cognitive and metacognitive strategies they use, etc.). The ultimate goal will be to propose instructional programmes that support intra- and intergroup functioning.

## Conclusions

This work has focused on studying different teaching modalities based on the use of active methodologies applied in Moodle and their relationship with student behaviour on the platform, learning outcomes and satisfaction with the teaching process. It has been found that although the teaching modalities include active methodologies, they do not have the same results in terms of behavioural profiles or student learning outcomes (Cabero-Almenara and Llorente-Cejudo, 2020). This is a significant element for reflection, since these differences may be due to various factors related to the students' own characteristics, such as digital competences (García-Peñalvo, 2021), cognitive, metacognitive, affective strategies, etc. (Bártolo-Ribeiro et al., 2020; Cloude et al., 2019; Wiedbusch et al., 2021; Yilmaz et al., 2020; Yoon et al., 2021), learning style and their response to Self-regulated learning (Valadas et al., 2017) or teacher characteristics, also related to digital competences and teaching style. These aspects will be addressed in future studies. Another relevant aspect to be studied in future work is the relationship dynamics within the collaborative groups; it has been detected that the interaction dynamics of the participants is not homogeneous. Similarly, another relevant element to be studied in detail is the use of visualisation resources for monitoring students (Álvarez-Arana et al., 2020; García-Peñalvo, 2020a; Verbert et al., 2013), such as UBUMonitor. These tools facilitate the functional monitoring of students in the LMS and allow the teacher to detect students who do not have a continuous learning pattern. However, if the teacher wishes to apply more complex techniques such as Machine Learning, it also facilitates their implementation (Vázquez-Ingelmo et al., 2021). In this study, it was found that the level of student performance and satisfaction was high, although not homogeneous, in the three teaching modalities applied. This conclusion shows that active methodologies are a good vehicle to encourage participation in virtual platforms and to achieve deeper learning (Chen et al., 2020; De Kock, 2016; García-Peñalvo, 2020a; Noroozi et al., 2019). Nevertheless, there are several factors that are influencing the results to be inconsistent. Therefore, directing research towards the detection of these factors is a relevant task for the twenty-first century society. In short, just the use of innovative methodologies applied in virtual platforms does not ensure learning success for all students (Agredo-Delgado et al., 2020). Among the possible factors that may explain this fact, the digital competence of the teacher and the student play an important role. Therefore, fostering the training of teachers and students (García-Peñalvo et al., 2020) is an important challenge for Higher Education institutions.

To sum up, the promotion of this type of teaching together with monitoring throughout the learning process is essential to achieve a more sustainable and inclusive education as supported by the OECD (2019) and the European Commission in the 2030 Agenda. This is the challenge for teachers and educational leaders especially in the framework of Higher Education which is geared towards reducing drop-out and ensuring that students acquire competences that will enable them to work effectively and successfully on graduation.

## Limitations and future work

The limitations of this work are related to the selection of the sample, since for ethical reasons it was not possible to randomise the groups to the different teaching modalities. Also, the sample is specific to third-year Health Science students. In addition, the composition of the groups was 3–6 participants and was not tested with more members, so the generalisation of the results should be made with caution. However, future studies will extend the type of participants to other areas of knowledge and in different academic years. Within we will check whether there are differences in behavioural profiles according to the number of members in each collaborative group (3, 4, 5, etc.).
